# First experiences with ^177^Lu-PSMA-617 therapy for recurrent or metastatic salivary gland cancer

**DOI:** 10.1186/s13550-021-00866-8

**Published:** 2021-12-14

**Authors:** Thomas J. W. Klein Nulent, Robert J. J. van Es, Stefan M. Willems, Arthur. J. A. T. Braat, Lot A. Devriese, Remco de Bree, Bart de Keizer

**Affiliations:** 1 Department of Head and Neck Surgical Oncology, University Medical Center Utrecht, P.O. Box 85500, Heidelberglaan 100, 3508 GA Utrecht, The Netherlands; 2 Department of Oral and Maxillofacial Surgery, University Medical Center Utrecht, Utrecht, The Netherlands; 3 Department of Pathology, University Medical Center Utrecht, Utrecht, The Netherlands; 4 Department of Pathology and Medical Biology, University Medical Center Groningen, Groningen, The Netherlands; 5 Department of Radiology and Nuclear Medicine, University Medical Center Utrecht, Utrecht, The Netherlands; 6 Department of Medical Oncology, University Medical Center Utrecht, Utrecht, The Netherlands

**Keywords:** Lutetium, Positron-emission tomography, Radionuclide, Adenoid cystic carcinoma, Salivary gland cancer

## Abstract

**Background:**

Advanced salivary gland cancers become difficult to treat when they are technically irresectable and radiotherapy limits are exceeded. There is also an unmet need to improve palliative systemic therapy. Salivary glands depict the Prostate-Specific Membrane Antigen (PSMA) on ^68^Ga-PSMA-PET/CT, a transmembrane protein that is targeted for diagnosis and treatment of advanced prostate cancer. Some salivary gland carcinomas also express PSMA.

**Methods:**

This study aimed to retrospectively evaluate the effectiveness of ^177^Lu-PSMA-617 therapy for recurrent or metastatic salivary gland cancers, as a last resort treatment. Patients with serious tumour-related discomfort for whom no regular option was available were selected and critically re-assessed by the tumour board. Radionuclide therapy eligibility was confirmed when tumour targeting was greater than liver SUVmax on ^68^Ga-PSMA-PET/CT. The protocol aimed at four cycles of 6.0–7.4 GBq ^177^Lu-PSMA-617 every 6–8 weeks. Clinical response was evaluated by questionnaires and radiological response by ^68^Ga-PSMA-PET/CT.

**Results:**

Six patients were treated with ^177^Lu-PSMA: four adenoid cystic carcinomas, one adenocarcinoma NOS and one acinic cell carcinoma. In two patients, radiological response was observed, showing either stable disease or a partial response, and four patients reported immediate relief of tumour-related symptoms. Most reported side effects were grade 1–2 fatigue, nausea, bone pain and xerostomia. Four patients prematurely discontinued therapy: three due to disease progression and one due to demotivating (grade 1) side-effects.

**Conclusions:**

Palliative ^177^Lu-PSMA therapy for salivary gland cancer may lead to rapid relief of tumour-associated discomfort and may even induce disease stabilization. It is safe, relatively well tolerated and can be considered when regular treatment options fail.

## Introduction

Salivary gland cancer is a rare malignant head and neck tumour. They account for 3–10% of all head and neck malignancies and exhibit a diverse clinical and biological behaviour. Adenoid cystic carcinoma (AdCC) is one of the most common malignant salivary gland tumours, comprising 20–35% of all cases [1, 2]. After initial treatment with surgery and often radiotherapy, patients with advanced disease are frequently confronted with a locoregional recurrence, as reflected by a local control rate of 58% after 10 years. Curation of progressive disease is challenging when a deep recurrence is technically irresectable and radiation limits are exceeded. Additionally, almost half of the patients develop slowly growing pulmonary or osseous distant metastases within 5 years after diagnosis that shorten life expectancy [1, 3–5]. Disease-specific survival (DSS) is moderate with 5- and 10-year survival rates of 68–78% and 54–65%, respectively [5, 6].

The effectiveness of both systemic chemotherapy and targeted immunotherapy is limited for symptomatic recurrent or distant disease and might only be beneficial to a small group of patients [7, 8].

The prostate-specific membrane antigen (PSMA), a transmembrane glycoprotein of the prostate secretory acinar epithelium, is known from its widely adopted use in diagnostics for metastatic prostate carcinoma using ^68^Ga-PSMA-PET/CT [9]. Research revealed tracer accumulation on ^68^Ga-PSMA-PET/CT not only in normal salivary and lacrimal glands, but also in areas of adenomas and adenocarcinomas such as AdCC, and more recently also in salivary duct carcinoma (SDC). Although intracellular PSMA expression was confirmed by histopathology in AdCC and in tumour-induced vessels of SDC, it is in general not correlated to PSMA-ligand uptake on PET/CT [9–13].

Palliative targeted radionuclide therapy with Lutetium-177 labelled PSMA-617 (^177^Lu-PSMA) is increasingly used in metastatic castration-resistant prostate cancer and well-tolerated with few side effects [14, 15]. Because of the clear visualization of AdCC and possible other salivary gland cancer localizations on PSMA PET/CT, it is of interest whether palliative salivary gland cancer patients could benefit from targeted therapy with ^177^Lu-PSMA, when other treatment options fail [12].

## Methods

Since mid-2018, the head and neck multidisciplinary tumour board (MTB) of the University Medical Center Utrecht offers compassionate use of the PSMA-targeted radionuclide ^177^Lu-PSMA therapy for patients with recurrent or metastatic salivary gland malignancies, as a last resort treatment. All consecutive patients who received this therapy until January 2021 were retrospectively analysed in this study.

Patients with increasing tumour-related discomfort, either during active follow-up or referred for a second opinion, deemed irresectable and without other standard palliative treatment options, were re-assessed at MTB and considered for ^177^Lu-PSMA therapy. Patient’s tumour PSMA-status was assessed in two ways: by analysing the tumour tissue PSMA expression by immunohistochemistry and by assessing PSMA-ligand uptake on ^68^Ga-PSMA-PET/CT, as described previously [12]. Sufficient PSMA ligand uptake assumed effective for ^177^Lu-PSMA targeting, was defined as a site of recurrent or metastatic disease with tracer uptake greater than normal liver uptake. When these conditions were met, patients were considered eligible for this radionuclide therapy.

### Treatment protocol

Patients were informed that the applied treatment was non-standard and involved the administration of a —for this application— non-registered radiopharmaceutical in a compassionate use program. Treatment aimed at four cycles of intravenous administration of 6.0–7.4 GBq ^177^Lu-PSMA-617 with an interval of 6–8 weeks. Prior to the start and during treatment, all tumour-related symptoms were accurately recorded. Moreover, laboratory red and white blood cell counts were obtained at all visits. All relevant treatment-related clinical and haematological adverse events were graded using the Common Terminology Criteria for Adverse Events (CTCAE) criteria, version 5.0.

For response evaluation a ^68^Ga-PSMA-PET/CT was performed. PET-CT was acquired from skull vertex to the thighs using a TruePoint Biograph mCT40 scanner (Siemens, Erlangen, Germany). A low dose CT scan was performed using Care Dose 4D and Care kV, reference parameters: 40 mAs, 120 kV. Subsequently, PET was acquired according to the European Association of Nuclear Medicine recommendations with the following parameters: PET with time-of-flight and point spread function (TrueX) reconstruction, 4 iterations, 21 subsets, with a filter of 7.5 mm full width at half maximum [16].

Evaluation was performed after each two cycles by ^68^Ga-PSMA-PET/CT [12]. SUVmax of the most accumulating lesion was measured on both the pre- and post-treatment scans using a freehand iso-contour volume of interest and lean body mass corrected formula. Response was defined as complete when all tumour localizations disappeared, as partial when SUVmax decreased ≥ 30%, as stable disease when there was neither a partial response nor progressive disease; and as progressive disease when SUVmax or tumour volume increased ≥ 20% or when new lesion(s) were discovered. In case of clinical or radiological progression of disease, the radionuclide treatment was discontinued.

## Results

**Fig. 1:**
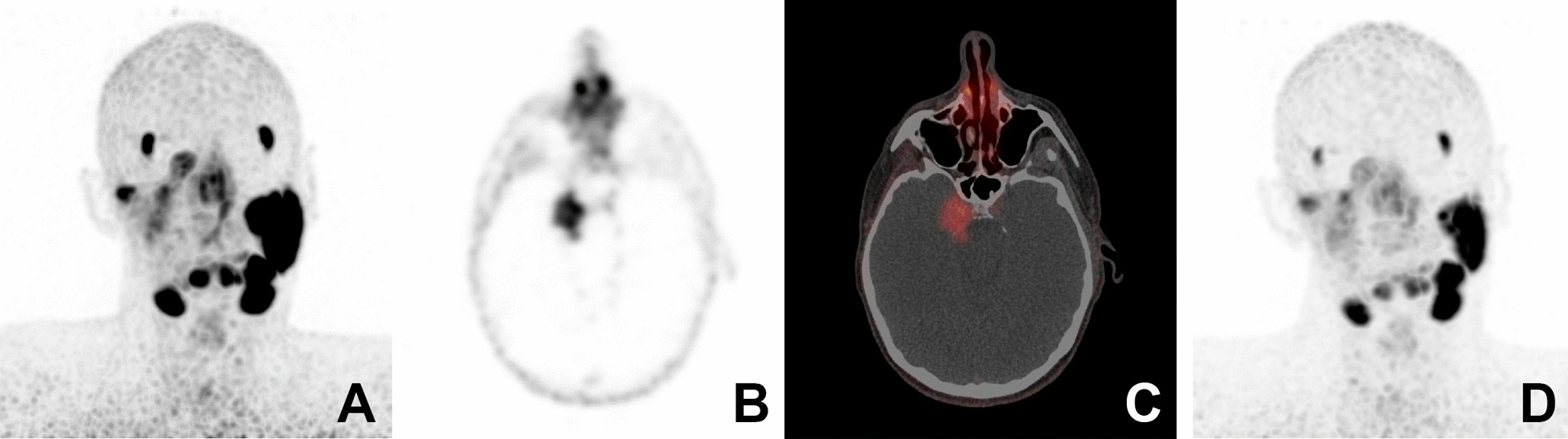
Patient no. 2 suffering from recurrent and metastatic AdCC of the right parotid gland. Imaging depicts moderate PSMA-ligand uptake in the recurrent parapharyngeal and intracranial tumour localizations: before (**A**, **B** and **C**) and after (**D**) therapy. **A** Coronal maximum intensity projection (before therapy SUVmax 6.5); **B** axial PET; **C** axial PET/CT reconstruction; **D** coronal maximum intensity projection (after therapy SUVmax 4.5)

Six patients were treated with ^177^Lu-PSMA of which four were diagnosed with AdCC, one with salivary gland adenocarcinoma (not otherwise specified) and one with acinic cell carcinoma. The tumours originated in the parotid gland in three, in the minor salivary glands of the oral cavity in two and in the submandibular gland in one patient. Further details are shown in Table 1. At the time of analysis, five of these six patients had died due to the disease, median 6 months after the start of the therapy. Targeting of the ^177^Lu-PSMA ligand was on average moderate and the mean SUVmax of patient’s most accumulating lesions was 8.2 (range 3.5–12.5; Fig. 1).

**Table 1:** Patient, disease and treatment characteristics

	Pt 1	Pt 2	Pt 3	Pt 4	Pt 5	Pt 6
Sex	F	M	M	F	M	F
Age at diagnosis	56	41	32	74	32	39
Year of diagnosis	2005	2013	2006	2016	2004	2017
Tumour type	AdCC	AdCC	AdCC	Adenocarcinoma NOS	Acinic cell carcinoma	AdCC
Tumour site	Hard palate	Parotid	Cheek mucosa	Parotid	Parotid	Submandibular gland
Treatment	Local excision	Local excision + radiotherapy	Local excision + radiotherapy	Palliative radiotherapy	Local excision + radiotherapy	Local excision + radiotherapy
*Disease*						
Locoregional recurrence	–	Parapharyngeal, intracranial	–	–	–	Parapharyngeal, lymphatic
Distant metastases	Lung, Liver	-	Lung, Bone (vertebra)	Bone (skull, vertebra)	Lymphatic (inguinal), Lung, Bone (vertebra)	-
Completed ‘conventional’ palliative treatment	None	None	Chemotherapy (CAP), radiotherapy	Radiotherapy	Chemotherapy (CAP), radiotherapy	Chemotherapy (CAP), radiotherapy
PSMA expression on IHC (%)	5	30	N/A	30	95	30
SUVmax VOI before treatment	3.5 lung	6.5 intracranial	10.2 pelvis	12.5 pelvis	9.7 pelvis	7.0 parapharyngeal
*Treatment*						
Diagnosis to ^177^Lu-PSMA (years)	12	6	12	1	14	2
No. cycles	4	4	2	1	2	2
SUVmax VOI after treatment	N/A	4.5	N/A	N/A	17.6	9.4
Reason of discontinuation	End of protocol	End of protocol	Disease progression, adverse effects	Demotivation due to side-effects	Disease progression	Disease progression
*Side effects*						
Side effects (CTCAE grade)	Fatigue (2) Dyspnoea (2) Nausea (1)	Fatigue (1) Nausea (1) Vomiting (1) Xerostomia (1)	Fatigue (1) Bone pain (2) Thrombocytopenia (3)	Fatigue (1) Xerostomia (1)	Fatigue (1) Xerostomia (1) Bone pain (2)	Fatigue (1) Xerostomia (2)
*Response*						
Clinical	Less dyspnoea, less fatigue	Improved facial expression and sensibility, less fatigue	Disease progression	N/A	Significant pain relief (6 weeks)	Diminution of facial nerve palsy, pain relief
Radiological	Stable lung lesions, minimal progression of liver metastases	Stable disease, decrease SUVmax	Disease progression	N/A	Disease progression	Disease progression
Follow-up (months after first treatment)	Deceased (7)	Alive with disease (36)	Deceased (3)	Deceased (5)	Deceased (6)	Deceased (9)

AdCC: Adenoid cystic carcinoma; CAP: cyclophosphamide, adriamycin, cisplatin; PSMA: prostate-specific membrane antigen; IHC: immunohistochemistry; N/A: not available; CTCAE: common terminology criteria for adverse events

Two patients completed the full study protocol of four cycles (no. 1 and 2). Patient no. 1 showed radiological stable disease of its lung metastases during the full treatment. The ^68^Ga-PSMA-PET/CT of patient no. 2 depicted a SUVmax decrease of 30% in the area of the recurrence that was classified as partial response, disease stabilization was up to 10 months after the start of the treatment —three months after the last cycle— when tumour growth was seen on ^68^Ga-PSMA-PET/CT.

Four patients reported subjective response by clear relief of tumour symptoms within the first weeks after the first cycle. The most common improvement was reduction in pain, followed by decrease in fatigue, less dyspnoea and improvement of facial expression by diminution of facial nerve palsy.

The therapy was well-tolerated in all cases except patient no. 3, who developed severe grade 3 thrombocytopenia (25.0–50.0 × 10e9/L) that led to discontinuation of the treatment, a possible adverse effect. There were no treatment-related deaths. Common side effects that were reported were fatigue, nausea, bone pain and xerostomia (graded CTCAE 1–2). Due to increase in fatigue and xerostomia, patient no. 4 was no longer motivated to continue the therapy after one cycle.

Immunohistochemical expression of PSMA showed similar expression patterns within the neoplastic cells of the different tumour subtypes and ranged from 5 to 95%. Within this small study population there was no correlation between pre-treatment SUVmax and tumour tissue PSMA expression. Patients no. 5 and 6 depicted the highest PSMA expression on immunohistochemistry (95% and 30%, respectively) and had a good initial clinical response. However, the intermediate ^68^Ga-PSMA-PET/CT after two cycles showed disease progression and the therapy was discontinued.

## Discussion

This study describes our first experiences with ^177^Lu-PSMA as palliative radionuclide treatment for recurrent and/or metastatic salivary gland malignancies of the head and neck. When regular palliative options fail, the present case series demonstrate that patients with sufficient tumour targeting on ^68^Ga-PSMA-PET/CT can be treated with PSMA-targeted radioligand therapy using ^177^Lu-PSMA, that may result in temporary radiological disease stabilization and relief of tumour-related discomfort. Two out of six patients showed disease stabilization for approximately 6 and 10 months respectively, four patients reported immediate reduction in symptoms, and disease progression was seen in three patients.

^177^Lu-PSMA therapy was generally well-tolerated and most side effects mentioned in this study are CTCAE graded 1–2. One patient was not motivated to continue treatment due to cumulative (grade 1) side-effects, and one patient developed severe (grade 3) thrombocytopenia that caused discontinuation of treatment. However, this adverse effect was more likely related to progressive bone-marrow metastases, as haemoglobin and leukocyte counts were severely suppressed as well.

In prostate cancer, palliative treatment with ^177^Lu-PSMA is known to achieve a prolonged progression-free survival and is considered safe. Hofman et al. described the results of this therapy in a selected group of 30 patients with intense PSMA-ligand binding on PET/CT and almost all (97%) of these patients showed biochemical response. Three months after a maximum of four cycles, a response evaluation was executed by ^68^Ga-PSMA-PET/CT: complete or partial radiological response was observed in, respectively, 10% and 30% and disease progression was reported in 57% of the patients. A large meta-analysis incorporated these results in their pooled analyses of 175 patients and reported even higher rates: 37% partial response, 38% stable disease and 25% progressive disease [17]. The recently published randomized VISION trial concluded a median increase in progression-free survival from 3.4 to 8.7 months, and an increased overall survival from 11.3 to 15.3 months when ^177^Lu-PSMA therapy was added to standard care [15].

Although both the treatment protocol and the administered activity in the present study were equal to the prostate cancer treatment schedules, response rates were less favourable: one out of six patients obtained a partial response on ^68^Ga-PSMA-PET/CT, and one patient had stable disease of lung metastases.

Remarkably, these two patients were the only two that completed all intended four cycles, whereas much more prostate cancer patients completed the full study protocol. The VISION trial even reported a median of five cycles per patient [9, 14, 15]. Another difference that may explain the less objective response of ^177^Lu-PSMA in salivary gland cancers is probably the lower PSMA-ligand uptake in the salivary gland tumours compared to prostate cancer lesions: a mean SUVmax 8.2 (range 3.5–12.5) versus 13.3 (range 0.7–122.5).

Clinical improvements and side-effects presented by this study are comparable to those mentioned in large prostate cancer radionuclide therapy reports: validated questionnaires revealed significant decrease in pain (≥ 1 point on Brief Pain Inventory pain severity score) and increased quality of life (≥ 10 points increase in EORTC QLQ-C30 global health score) in approximately half of the patients after two cycles [14, 18]. Most reported side effects in these reports were fatigue (43%), dry mouth (39%), nausea (35%), bone-marrow depression (32%) and back pain/arthralgia (23%); loss of appetite, diarrhoea or constipation, vomiting and nephrotoxicity were less common [15, 17].

First studies on radionuclide therapy for other solid tumours that express PSMA have recently been summarized [19]. When compared to glioblastoma, thyroid carcinoma, renal cell carcinoma and hepatocellular carcinoma that all express PSMA in the tumour’s neovasculature and not intracellular, ligand uptake in salivary gland tumours is moderate to weak. Further research should reveal whether salivary gland cancer patients could benefit from a higher dose or shortened interval of ^177^Lu-PSMA. Furthermore, Alpha-emitting agents such as ^225^Ac-PSMA-617 may be more successful due to their higher linear energy transmission [20].

Until now, a few studies report on PSMA related imaging and/or therapy in salivary gland tumours. We previously described visualization of local recurrent and distant metastatic AdCC on ^68^Ga-PSMA-PET/CT, and confirmed the PSMA-specific targeting of these tumours by high intratumoural PSMA-expression on immunohistochemistry [12, 21]. Only one case report has been published regarding radionuclide therapy in metastatic AdCC of the parotid. ^68^Ga-PSMA uptake was seen in all sites of known AdCC bone metastases and subsequently ^177^Lu-PSMA was administered after all regular treatment options failed. The post-therapy imaging showed intense tracer uptake and the patient reported immediate significant pain relief in the weeks after the therapy. Due to severe hypercalcaemia the treatment was discontinued after one cycle, an adverse effect that we did not encounter before [22]. Although a recent study focussed on PSMA-ligand uptake in SDC, there are no other reports that support the results of the present study [13].

Clinical response that combines decrease in tumour-related discomfort with only limited toxic side effects is of importance in palliative treatment. In our opinion, the current therapy positively affects quality of life and meets the criteria of a noteworthy palliative option [23].

## Conclusion

When tumour targeting is sufficient, palliative PSMA-targeted radioligand therapy of advanced or metastasized salivary gland cancers with ^177^Lu-PSMA may cause a significant relief of tumour-associated discomfort in the majority of the patients, and may induce a partial response or even stable disease in one-third of the cases. The presented protocol is safe and relatively well-tolerated. When regular treatment options fail, indicative targeted imaging using ^68^Ga-PSMA-PET/CT can be considered to assess eligibility for ^177^Lu-PSMA therapy.

## Data Availability

The datasets generated and/or analysed during the current study are not publicly available, but are available from the corresponding author on reasonable request.

## Abbreviations

PSMA: Prostate-specific membrane antigen
AdCC: Adenoid cystic carcinoma
SDC: Salivary duct carcinoma
MTB: Multidisciplinary tumour board
CTCAE: Common terminology criteria for adverse events
